# Supplementary material for: Mathematical modeling to reveal breakthrough mechanisms in the HIV Antibody Mediated Prevention (AMP) trials
Source: PLoS Comput Biol. 2020 Feb 21;16(2):e1007626. doi: 10.1371/journal.pcbi.1007626 (PMC7055956; doi:10.1371/journal.pcbi.1007626)
Supplement: Attachment — Submitted filename: AMP model R0.nb.pdf [file pcbi.1007626.s004.pdf]

# Calculating $R_0$ for the AMP model of HIV infection

In[110]:= `Clear["`*"];`

## Define equations

(*Note that E is a defined function in Mathematica so use Ee instead)

In[111]:=
```
eqS = αS - δS * S[t] - S[t] * V[t] * (1 - v) * β
eqIap = β * τ * (1 - λ) * (1 - v) * V[t] * S[t] - δI * Iap[t] - κ * Ee[t] * Iap[t]
eqIau = β * (1 - τ) * (1 - λ) * (1 - v) * V[t] * S[t] - δI * Iau[t] - κ * Ee[t] * Iau[t]
eqIlp = β * τ * λ * (1 - v) * V[t] * S[t] - δL * Ilp[t]
eqIlu = β * (1 - τ) * λ * (1 - v) * V[t] * S[t] - δL * Ilu[t]
eqEe = αE + ω * (Iap[t] + Iau[t]) * Ee[t] / (Ee[t] + EC50) - δE * Ee[t]
eqV = π * Iap[t] - γ * V[t] - β * (1 - v) * S[t] * V[t]
```

Out[111]= $\alpha_S - S[t]\,\delta_S - (1-v)\,\beta\,S[t]\,V[t]$

Out[112]= $-\kappa\,Ee[t]\,Iap[t] - Iap[t]\,\delta_i + (1-v)\,\beta\,(1-\lambda)\,\tau\,S[t]\,V[t]$

Out[113]= $-\kappa\,Ee[t]\,Iau[t] - Iau[t]\,\delta_i + (1-v)\,\beta\,(1-\lambda)\,(1-\tau)\,S[t]\,V[t]$

Out[114]= $-Ilp[t]\,\delta_L + (1-v)\,\beta\,\lambda\,\tau\,S[t]\,V[t]$

Out[115]= $-Ilu[t]\,\delta_L + (1-v)\,\beta\,\lambda\,(1-\tau)\,S[t]\,V[t]$

Out[116]= $\frac{\omega\,Ee[t]\,(Iap[t]+Iau[t])}{Ee[t]+EC_{50}} + \alpha_e - Ee[t]\,\delta_e$

Out[117]= $\pi\,Iap[t] - \gamma\,V[t] - (1-v)\,\beta\,S[t]\,V[t]$

## Find equilibrium values

Calculate the uninfected equilibrium

In[118]:=
```
equilibria0 = Solve[
  {eqS == 0, eqIap == 0, eqIau == 0, eqIlp == 0, eqIlu == 0, eqEe == 0, eqV == 0} /. {V[t] → 0},
  {S[t], Iap[t], Iau[t], Ilp[t], Ilu[t], Ee[t]}
 ]
```

Out[118]= $\left\{\left\{S[t] \to \frac{\alpha_S}{\delta_S},\ Iap[t] \to 0,\ Iau[t] \to \frac{(\kappa\,EC_{50} - \delta_i)\,(\kappa\,\alpha_e + \delta_i\,\delta_e)}{\kappa\,\omega\,\delta_i},\ Ilp[t] \to 0,\ Ilu[t] \to 0,\ Ee[t] \to -\frac{\delta_i}{\kappa}\right\},\ \left\{S[t] \to \frac{\alpha_S}{\delta_S},\ Iap[t] \to 0,\ Iau[t] \to 0,\ Ilp[t] \to 0,\ Ilu[t] \to 0,\ Ee[t] \to \frac{\alpha_e}{\delta_e}\right\}\right\}$

(*only the second equilibrium exists, since all variables must be positive)

In[119]:= `equilibria0[[2]]`

Out[119]= $\left\{S[t] \to \frac{\alpha_S}{\delta_S}, Iap[t] \to 0, Iau[t] \to 0, Ilp[t] \to 0, Ilu[t] \to 0, Ee[t] \to \frac{\alpha_e}{\delta_e}\right\}$

In[120]:= `eq0 = Append[equilibria0[[2]], V[t] → 0]`

Out[120]= $\left\{S[t] \to \frac{\alpha_S}{\delta_S}, Iap[t] \to 0, Iau[t] \to 0, Ilp[t] \to 0, Ilu[t] \to 0, Ee[t] \to \frac{\alpha_e}{\delta_e}, V[t] \to 0\right\}$

## Calculate R0 using the next generation matrix

This calculation follows the methods outlined in the review paper: Heffernan JM, Smith RJ, Wahl LM. Perspectives on the basic reproductive ratio. J Roy Soc Interface (2005) 2, 281-293

Collect terms that involve the create of new infections (rate of new infections in each cell compartment) - F matrix

In[121]:=
```
FS = 0
FIap = β * τ * (1 - λ) * (1 - v) * V[t] * S[t]
FIau = β * (1 - τ) * (1 - λ) * (1 - v) * V[t] * S[t]
FIlp = β * τ * λ * (1 - v) * V[t] * S[t]
FIlu = β * (1 - τ) * λ * (1 - v) * V[t] * S[t]
FEe = 0
FV = π * Iap[t]
```

Out[121]= 0

Out[122]= $(1 - v)\, \beta\, (1 - \lambda)\, \tau\, S[t]\, V[t]$

Out[123]= $(1 - v)\, \beta\, (1 - \lambda)\, (1 - \tau)\, S[t]\, V[t]$

Out[124]= $(1 - v)\, \beta\, \lambda\, \tau\, S[t]\, V[t]$

Out[125]= $(1 - v)\, \beta\, \lambda\, (1 - \tau)\, S[t]\, V[t]$

Out[126]= 0

Out[127]= $\pi\, Iap[t]$

Calculate the Jacobian of the F matrix, at the uninfected equilibrium

In[128]:= `vars = {S[t], Iap[t], Iau[t], Ilp[t], Ilu[t], Ee[t], V[t]}`

Out[128]= {S[t], Iap[t], Iau[t], Ilp[t], Ilu[t], Ee[t], V[t]}

In[129]:= `Feqs = {FS, FIap, FIau, FIlp, FIlu, FEe, FV}`

Out[129]= $\{0, (1 - v)\, \beta\, (1 - \lambda)\, \tau\, S[t]\, V[t], (1 - v)\, \beta\, (1 - \lambda)\, (1 - \tau)\, S[t]\, V[t], (1 - v)\, \beta\, \lambda\, \tau\, S[t]\, V[t], (1 - v)\, \beta\, \lambda\, (1 - \tau)\, S[t]\, V[t], 0, \pi\, Iap[t]\}$

In[130]:=
```
DF = D[Feqs, {vars}];
DF // MatrixForm
```

Out[131]//MatrixForm=

$$\begin{pmatrix} 0 & 0 & 0 & 0 & 0 & 0 & 0 \\ (1-v)\,\beta\,(1-\lambda)\,\tau\,V[t] & 0 & 0 & 0 & 0 & 0 & (1-v)\,\beta\,(1-\lambda)\,\tau\,S[t] \\ (1-v)\,\beta\,(1-\lambda)\,(1-\tau)\,V[t] & 0 & 0 & 0 & 0 & 0 & (1-v)\,\beta\,(1-\lambda)\,(1-\tau)\,S[t] \\ (1-v)\,\beta\,\lambda\,\tau\,V[t] & 0 & 0 & 0 & 0 & 0 & (1-v)\,\beta\,\lambda\,\tau\,S[t] \\ (1-v)\,\beta\,\lambda\,(1-\tau)\,V[t] & 0 & 0 & 0 & 0 & 0 & (1-v)\,\beta\,\lambda\,(1-\tau)\,S[t] \\ 0 & 0 & 0 & 0 & 0 & 0 & 0 \\ 0 & \pi & 0 & 0 & 0 & 0 & 0 \end{pmatrix}$$

In[132]:=
```
DF0 = Simplify[DF /. eq0];
DF0 // MatrixForm
```

Out[133]//MatrixForm=

$$\begin{pmatrix} 0 & 0 & 0 & 0 & 0 & 0 & 0 \\ 0 & 0 & 0 & 0 & 0 & 0 & \frac{(-1+v)\,\beta\,(-1+\lambda)\,\tau\,\alpha_S}{\delta_S} \\ 0 & 0 & 0 & 0 & 0 & 0 & -\frac{(-1+v)\,\beta\,(-1+\lambda)\,(-1+\tau)\,\alpha_S}{\delta_S} \\ 0 & 0 & 0 & 0 & 0 & 0 & -\frac{(-1+v)\,\beta\,\lambda\,\tau\,\alpha_S}{\delta_S} \\ 0 & 0 & 0 & 0 & 0 & 0 & \frac{(-1+v)\,\beta\,\lambda\,(-1+\tau)\,\alpha_S}{\delta_S} \\ 0 & 0 & 0 & 0 & 0 & 0 & 0 \\ 0 & \pi & 0 & 0 & 0 & 0 & 0 \end{pmatrix}$$

Collect all other terms in equations (times -1) - this is the net rate of transfer out of that compartment by all other means - V matrix

In[134]:=
```
VS = - (αS - δS * S[t])
VIap = - (-δI * Iap[t] - κ * Ee[t] * Iap[t])
VIau = - (-δI * Iau[t] - κ * Ee[t] * Iau[t])
VIlp = - (-δL * Ilp[t])
VIlu = - (β - δL * Ilu[t])
VEe = - (αE + ω * (Iap[t] + Iau[t]) * Ee[t] / (Ee[t] + EC50) - δE * Ee[t])
VV = - (-γ * V[t] - β * (1 - v) * S[t] * V[t])
```

Out[134]= $-\alpha_S + S[t]\,\delta_S$

Out[135]= $\kappa\,Ee[t]\,Iap[t] + Iap[t]\,\delta_{i}$

Out[136]= $\kappa\,Ee[t]\,Iau[t] + Iau[t]\,\delta_{i}$

Out[137]= $Ilp[t]\,\delta_L$

Out[138]= $-\beta + Ilu[t]\,\delta_L$

Out[139]= $-\frac{\omega\,Ee[t]\,(Iap[t] + Iau[t])}{Ee[t] + EC_{50}} - \alpha_e + Ee[t]\,\delta_e$

Out[140]= $\gamma\,V[t] + (1-v)\,\beta\,S[t]\,V[t]$

Calculate the Jacobian of the V matrix, at the uninfected equilibrium

In[141]:=
```
Veqs = {VS, VIap, VIau, VIlp, VIlu, VEe, VV}
```

Out[141]=

$$\left\{-\alpha_S + S[t]\,\delta_S,\ \kappa\,Ee[t]\,Iap[t] + Iap[t]\,\delta_{i},\ \kappa\,Ee[t]\,Iau[t] + Iau[t]\,\delta_{i},\ Ilp[t]\,\delta_L,\ -\beta + Ilu[t]\,\delta_L,\ -\frac{\omega\,Ee[t]\,(Iap[t]+Iau[t])}{Ee[t]+EC_{50}} - \alpha_e + Ee[t]\,\delta_e,\ \gamma\,V[t] + (1-v)\,\beta\,S[t]\,V[t]\right\}$$

In[142]:=
```
DV = D[Veqs, {vars}];
DV // MatrixForm
```

Out[143]//MatrixForm=

$$\begin{pmatrix} \delta_S & 0 & 0 & 0 & 0 & 0 & \\ 0 & \kappa\,Ee[t]+\delta_{i} & 0 & 0 & 0 & \kappa\,Iap[t] & \\ 0 & 0 & \kappa\,Ee[t]+\delta_{i} & 0 & 0 & \kappa\,Iau[t] & \\ 0 & 0 & 0 & \delta_L & 0 & 0 & \\ 0 & 0 & 0 & 0 & \delta_L & 0 & \\ 0 & -\frac{\omega\,Ee[t]}{Ee[t]+EC_{50}} & -\frac{\omega\,Ee[t]}{Ee[t]+EC_{50}} & 0 & 0 & \frac{\omega\,Ee[t]\,(Iap[t]+Iau[t])}{(Ee[t]+EC_{50})^2} - \frac{\omega\,(Iap[t]+Iau[t])}{Ee[t]+EC_{50}} + \delta_e & \\ (1-v)\,\beta\,V[t] & 0 & 0 & 0 & 0 & 0 & \gamma + (1 - \end{pmatrix}$$

In[144]:=
```
DV0 = Simplify[DV /. eq0];
DV0 // MatrixForm
```

Out[145]//MatrixForm=

$$\begin{pmatrix} \delta_S & 0 & 0 & 0 & 0 & 0 & 0 \\ 0 & \delta_{i} + \frac{\kappa\,\alpha_e}{\delta_e} & 0 & 0 & 0 & 0 & 0 \\ 0 & 0 & \delta_{i} + \frac{\kappa\,\alpha_e}{\delta_e} & 0 & 0 & 0 & 0 \\ 0 & 0 & 0 & \delta_L & 0 & 0 & 0 \\ 0 & 0 & 0 & 0 & \delta_L & 0 & 0 \\ 0 & -\frac{\omega\,\alpha_e}{\alpha_e+EC_{50}\,\delta_e} & -\frac{\omega\,\alpha_e}{\alpha_e+EC_{50}\,\delta_e} & 0 & 0 & \delta_e & 0 \\ 0 & 0 & 0 & 0 & 0 & 0 & \gamma - \frac{(-1+v)\,\beta\,\alpha_S}{\delta_S} \end{pmatrix}$$

In[146]:=
```
DV0I = Inverse[DV0];
DV0I // MatrixForm
```

Out[147]//MatrixForm=

$$\begin{pmatrix} \frac{1}{\delta_S} & 0 & 0 & 0 & 0 & 0 & 0 \\ 0 & \frac{\kappa\,\alpha_e+\delta_{i}\,\delta_e}{\left(\delta_{i}+\frac{\kappa\,\alpha_e}{\delta_e}\right)^2\delta_e} & 0 & 0 & 0 & 0 & 0 \\ 0 & 0 & \frac{\kappa\,\alpha_e+\delta_{i}\,\delta_e}{\left(\delta_{i}+\frac{\kappa\,\alpha_e}{\delta_e}\right)^2\delta_e} & 0 & 0 & 0 & 0 \\ 0 & 0 & 0 & \frac{1}{\delta_L} & 0 & 0 & 0 \\ 0 & 0 & 0 & 0 & \frac{1}{\delta_L} & 0 & 0 \\ 0 & \frac{\frac{\omega\,\alpha_e\,\delta_{i}}{\alpha_e+EC_{50}\,\delta_e}+\frac{\kappa\,\omega\,\alpha_e^2}{\delta_e\,(\alpha_e+EC_{50}\,\delta_e)}}{\left(\delta_{i}+\frac{\kappa\,\alpha_e}{\delta_e}\right)^2\delta_e} & \frac{\frac{\omega\,\alpha_e\,\delta_{i}}{\alpha_e+EC_{50}\,\delta_e}+\frac{\kappa\,\omega\,\alpha_e^2}{\delta_e\,(\alpha_e+EC_{50}\,\delta_e)}}{\left(\delta_{i}+\frac{\kappa\,\alpha_e}{\delta_e}\right)^2\delta_e} & 0 & 0 & \frac{1}{\delta_e} & 0 \\ 0 & 0 & 0 & 0 & 0 & 0 & \frac{1}{\gamma-\frac{(-1+v)\,\beta\,\alpha_S}{\delta_S}} \end{pmatrix}$$

Multiple the matrices and calculate the spectral radius

In[148]:= `A = DF0.DV0I`

Out[148]=
$$\left\{\{0,0,0,0,0,0,0\},\left\{0,0,0,0,0,0,\frac{(-1+v)\,\beta\,(-1+\lambda)\,\tau\,\alpha_S}{\left(\gamma-\frac{(-1+v)\,\beta\,\alpha_S}{\delta_S}\right)\delta_S}\right\},\left\{0,0,0,0,0,0,-\frac{(-1+v)\,\beta\,(-1+\lambda)\,(-1+\tau)\,\alpha_S}{\left(\gamma-\frac{(-1+v)\,\beta\,\alpha_S}{\delta_S}\right)\delta_S}\right\},\left\{0,0,0,0,0,0,-\frac{(-1+v)\,\beta\,\lambda\,\tau\,\alpha_S}{\left(\gamma-\frac{(-1+v)\,\beta\,\alpha_S}{\delta_S}\right)\delta_S}\right\},\left\{0,0,0,0,0,0,\frac{(-1+v)\,\beta\,\lambda\,(-1+\tau)\,\alpha_S}{\left(\gamma-\frac{(-1+v)\,\beta\,\alpha_S}{\delta_S}\right)\delta_S}\right\},\{0,0,0,0,0,0,0\},\left\{0,\frac{\pi\,(\kappa\,\alpha_e+\delta_i\,\delta_e)}{\left(\delta_i+\frac{\kappa\,\alpha_e}{\delta_e}\right)^2\delta_e},0,0,0,0,0\right\}\right\}$$

In[149]:= `eA = Eigenvalues[A]`

Out[149]=
$$\left\{0,0,0,0,0,-\frac{i\sqrt{\pi}\,\sqrt{-1+v}\,\sqrt{\beta}\,\sqrt{-1+\lambda}\,\sqrt{\tau}\,\sqrt{\alpha_S}\,\sqrt{\delta_e}}{\sqrt{\kappa\,\alpha_e+\delta_i\,\delta_e}\,\sqrt{-\beta\,\alpha_S+v\,\beta\,\alpha_S-\gamma\,\delta_S}},\frac{i\sqrt{\pi}\,\sqrt{-1+v}\,\sqrt{\beta}\,\sqrt{-1+\lambda}\,\sqrt{\tau}\,\sqrt{\alpha_S}\,\sqrt{\delta_e}}{\sqrt{\kappa\,\alpha_e+\delta_i\,\delta_e}\,\sqrt{-\beta\,\alpha_S+v\,\beta\,\alpha_S-\gamma\,\delta_S}}\right\}$$

In[150]:= `R0 = eA[[6]]^2`

Out[150]=
$$-\frac{\pi\,(-1+v)\,\beta\,(-1+\lambda)\,\tau\,\alpha_S\,\delta_e}{(\kappa\,\alpha_e+\delta_i\,\delta_e)\,(-\beta\,\alpha_S+v\,\beta\,\alpha_S-\gamma\,\delta_S)}$$
