# Supplementary material for: Mathematical modeling to reveal breakthrough mechanisms in the HIV Antibody Mediated Prevention (AMP) trials
Source: PLoS Comput Biol. 2020 Feb 21;16(2):e1007626. doi: 10.1371/journal.pcbi.1007626 (PMC7055956; doi:10.1371/journal.pcbi.1007626)
Supplement: Attachment — Submitted filename: Derivation.pdf [file pcbi.1007626.s006.pdf]

Calculating R_0 for the AMP HIV model

September 27, 2019

The model is described by the following system of differential equations

$$\begin{aligned}
\dot{S} &= \alpha_S - \delta_S S - \beta(1-v)SV \\
\dot{I}_{A,P} &= \beta(1-v)\tau(1-\lambda)VS - (\delta_I + \kappa E)I_{A,P} \\
\dot{I}_{A,U} &= \beta(1-v)(1-\tau)(1-\lambda)VS - (\delta_I + \kappa E)I_{A,U} \\
\dot{I}_{L,P} &= \beta(1-v)\tau\lambda VS - \delta_L I_{L,p} \\
\dot{I}_{L,U} &= \beta(1-v)(1-\tau)\lambda VS - \delta_L I_{L,U} \\
\dot{E} &= \alpha_e + \omega(I_{A,p} + I_{A,U})\frac{E}{E + EC_{50}} - \delta_E E \\
\dot{V} &= \pi I_{A,P} - \gamma V - \beta(1-v)SV
\end{aligned} \tag{1}$$

The definition of R_0 is the average number of secondary infections infections produced by a single infected cell over the course of its lifetime, when this is the only infected cell in the population (e.g. cells are otherwise fully susceptible). We interpret this to mean the R_0 experienced by the first infected cell after a transmission event, and assume that at the time of infection, the population of cells is at the uninfected equilibrium level (all derivatives zero), e.g.

$$\begin{aligned}
S^* &= \frac{\alpha_S}{\delta_S} \\
{I^*}_{i,j} &= 0 \quad \text{for all} \quad i,j \\
E^* &= \frac{\alpha_e}{\delta_E} \\
V^* &= 0
\end{aligned} \tag{2}$$

Notice that in this system, the latently infected cells are a “dead end” .. they don’t produce virus and their levels do not influence the dynamics of any other cells. Active but unproductive cells also do not contribute to infection, since they produce no virions, though they do stimulate the adaptive immune response and therefore act to reduce the levels of active and productive cells, who are the only ones to produce virus. Consequently, in calculating R_0, the cell we need to track – the only one that can produce secondary infections — is $I_{A,P}$.

The average number of offspring produced per $I_{A,P}$ cell is the product of a) the average lifespan of an $I_{A,P}$ cell, b) the rate at which virus is produced from this cell, c) the average lifespan of these virions, d) the rate at which each virion infects susceptible cells to produce new infections, and e) the fraction of these infections that are active and productive. Together, this gives us the following expression for R_0

$$
\begin{aligned}
R_0 &= a * b * c * d * e \\
&= \frac{1}{(\delta_I + \kappa E^*)} \pi \frac{1}{(\delta + \beta(1-v)S^*)} \beta\tau(1-\lambda)S^* \\
&= \frac{\pi\beta\tau(1-\lambda)(1-v)\alpha_S}{\delta_S(\delta_I + \kappa\alpha_E/\delta_E)(\gamma + \beta(1-v)\alpha_S/\delta_S)} \\
&= \frac{\pi\beta\tau(1-\lambda)(1-v)\alpha_S}{\delta_I(1 + \kappa\alpha_E/\delta_I\delta_E)(\gamma\delta_S + \beta(1-v)\alpha_S)} \\
&\approx \frac{\pi\beta\tau(1-\lambda)(1-v)\alpha_S}{\delta_I\gamma\delta_S(1 + \kappa\alpha_E/\delta_I\delta_E)}
\end{aligned} \tag{3}
$$

where the approximation follows that in the text – assuming that viral production and binding of virus to antibody are much faster than loss of virus due to infection. This equation for R_0 is identical to that derived by the next-generation method in the Mathematica file.
