# Supplementary material for: Mathematical modeling to reveal breakthrough mechanisms in the HIV Antibody Mediated Prevention (AMP) trials
Source: PLoS Comput Biol. 2020 Feb 21;16(2):e1007626. doi: 10.1371/journal.pcbi.1007626 (PMC7055956; doi:10.1371/journal.pcbi.1007626)
Supplement: Attachment — Submitted filename: AMP_response.docx [file pcbi.1007626.s007.docx]

**Dear Editors Gallo/deBoer,**

**Enclosed is the second revised version of our manuscript “Mathematical Modeling to Reveal Breakthrough Mechanisms in the HIV Antibody Mediated Prevention (AMP) Trials”. We addressed each of the reviewers’ points in the letter below and made relevant changes in the manuscript. We attach a new clean version as well as a highlighted version indicating where edits were made.**

**We are grateful for the high quality of reviews and appreciate the feedback, which has substantially improved the reproducibility, quality and clarity of our paper.**

**Our responses are indented below in bold:**

Reviewer #1: In this study Reeves et al present a framework to predict and interpret the outcomes of trials for a new class of HIV therapeutics. These planned “Antibody mediated prevention” trials will test whether infrequent injections of synthetic anti-HIV antibodies can offer long-term prevention of new infections. This could be a paradigm shift in HIV prevention, since current prevention methods suffer from extreme difficulties in patient compliance (e.g. PrEP - taking antiretroviral pills daily as prophylaxis, or condom use, other behavioral modifications). There are two major open questions about these studies which Reeves et al address: What fraction of new infections are likely to be prevented by these antibodies and at what administered dose? And, if these trials are less successful than predicted, how can we understand what was the mechanism for failure?

Reeves et al develop and calibrate a modeling framework which includes a) pharmacokinetics of antibody in the blood, b) dose-dependent neutralizing potency of antibodies across a spectrum of different viral strains, c) the dynamics of acute HIV infection and its predicted dependence on % of free virus bound by antibody, and d) the frequency of HIV transmission and its predicted dependence on antibody administration at a population level. Overall the paper is a beautiful example of how modeling an integrate pre-clinical data from multiple sources to make informed predictions about new resource-intensive trials, and, how models can also be used to extract the most possible knowledge from the outcomes of these trials - be they successes or failures. The paper is very clearly written, the models and other methods well-justified, and the results presented in an intuitive way.

One main result of the paper is that the prevention efficacy of 10 mg/kg of VCR01 antibody administered every 8 weeks is 83%, while a 30 mg/kg dose is expected to have 92% efficacy. The second major result is that the two most likely modes of suboptimal prevention efficacy can be distinguished at a population scale if the antibody neutralization potency is measured (in vitro) for the virus of all individuals who get infected despite being on the therapy. The authors identify the two most likely mechanisms to comprise study efficacy: Firstly, the antibody may have a lower efficacy in vivo compared to in vitro, against all viral strains. This could be due to suboptimal pharmacokinetics in tissue sites relevant to infection or due to antibody competition. Secondly, there could be specific viral strains that are circulating which are very resistant to the antibody, but haven’t properly been catalogued in the database of strains the authors use for their main predictions. They show that these cases will both cause a reduction in study efficacy, but that the nature of viral strains that end up causing infections despite therapy will be different. If overall low potency is at play, then failure can be caused by strains with low IC50 (not very resistant), and there will be lots of variation in the IC50 of the strains that "break through". However if there are more circulating resistant strains and this causes poor prevention efficacy in the study, then when individuals do become infected, it is more likely to be caused by these very resistant strains, and there will be less variation in the IC50 of break through strains (uniformly high IC50).

Overall I think this paper would make an excellent addition to PLOS Computational Biology. The results will be directly and immediately useful to the four AMP trials that are beginning soon, and, to the many others that will likely follow. In addition, their modeling framework could also be applied to other modalities of prevention.

I only have a few suggestions and questions about the model. Most of these are relatively minor, though one I think is relatively serious since it is a clear mistake in the mathematics and will have some influence on the results. However, I think it is very easy to fix and does not change at all the overall framework the authors use.

**Thank you sincerely for this summary and comments, we are indebted to your effort.**

Comments

1. R0 calculation: The formula for R0 that the authors give in the paper is wrong. This is caused by a fundamental misunderstanding of the underlying definition of R0. R0 generally provides the role of a threshold quantity in infection models, in that if R0<1 the infection will never take off or head towards extinction, whereas if R0>1 the infection will grow and reach a stable equilibrium where infection persists. However, there are infinitely many quantities that have this threshold behavior, but only one of them is equal to R0. There is only one specific expression that gives R0. R0 has the intuitive meaning as the average number of secondary infected cells produced by a single infected cell over the course of its lifespan, in a population that is otherwise at the uninfected equilibrium. In the author’s model, there are many types of cells, but since only the active/productive cells produce secondary infections, R0 should be the average number of secondary I_A,P cells produced from a single I_A,P cell. Many of these issues are discussed very clearly in the following paper: Heffernan JM, Smith RJ, Wahl LM, Perspectives on the basic reproductive ratio, JRSI, 2005. There are a few different ways to calculate R0 correctly and they will all give the same answer (different from the authors expression). I have attached documents showing both of these methods. The first (Derivation of R0.pdf), is often called the “survival function method” but is really just an accounting for the probabilities of survival and infection production throughout each stage of infection over the course of a generation. The second is the more formal next-generation matrix, and this calculation is done step by step in the attached Mathematica notebooks (Mathematica Output for R0.nb), from which I also printed off a pdf. Getting the expression for R0 right is very important for studies like this, because the impact that a given potency of therapy has on infection dynamics depends directly on how much it reduces R0 and whether that reduces R0 to less than one or not. If the wrong expression for R0 is used and R0 is assumed to be much smaller than it really is, predictions of treatment efficacy will be over optimistic. It is true that we can never be certain about the underlying model, but that is actually a bit tangential to the issue of estimating R0. At minimum R0 must be internally consistent within a model.

**We very much appreciate your work, and the extremely clear point about threshold quantities vs R0. We have amended our paper accordingly with a revised derivation based on your survival function. We should have checked the simple derivation in the last version against the next generation (or other formal) method to find the mistake. Fortunately, due to the parameter values, the new expression is numerically very similar to our initial expression. We also cite the reference you suggest, which is an excellent work.**

2. There is one second more minor issue related to R0. In general when estimating R0 from infection trajectories, R0 is determined from the early rate of exponential growth (r), and, importantly, the lifespan of infected cells (T). There is an intuitive explanation for this: the rate at which infection grows depends on how many offspring each infected cell is producing but also how long it takes to produce those offspring. In general there is some sort of expression like r ~ (R0-1)*/T. So what the authors say about their model being equally valid in its estimating of R0 to other models (e.g. Regoes 2010) despite different estimates of lifespan is simply not true. An accurate estimate of R0 requires an accurate estimate of lifespan. So R0 is only estimated correctly if the authors think their estimate of lifespan is correct.

**Apologies for our writing which led to these confusing statements. Our goal here was to illustrate that estimates of R0 are model specific – some in the field have absorbed Ribeiro et al.’s estimation and can be surprised when a different value of R0 is calculated. We have changed wording around line 95 to be more concrete.**

3. I was a bit confused by the terminology of “productive” vs “unproductive” infection. both of which can happen in either active or latently infected cells. Is this meant to refer to whether the viral genome is intact or defective? It’s hard to understand the way these cells behave in the model. How can active, unproductive cells stimulate the immune response (non-zero omega) but not produce free virus? And is it really necessary to track these cells?

**We introduced this compartment in Reeves et al. JVI 2017, because we found it was an extremely useful compartment to modulate the value of viral setpoint and fit diverse/heterogeneous SHIV viral loads. We described this term biologically as the creation of infected cells that do not ever go on to create further infected cells due to fatal defective mutations. It has been shown previously that such defective viral sequences can still engender an immune response – perhaps by making viral peptides that are noticed but not packaged correctly, and in any case without making functional virus. Since the writing of that paper, we have also recognized that these cells could be representing a different mechanism as well – bystander killing and therefore immune signaling that occurs simply due to cytokine activity during CTL action. In any case, we showed in the mentioned paper that models without this compartment, and even models with such a compartment but that did not also recruit adaptive immunity, achieved worse fit (by AIC). Therefore, we kept the term here, and it does indeed help fitting to human data.**

4. The latent compartment has no influence on the kinetics observed during acute infection and therefore on the fit model - might be clearer just to say this, and that these parameters must be estimated from other types of data (on decay of LR in patients on ART and based on known fraction cells latently infected). This is different from other parameters, like Beta, alpha_s , pi, gamma, which were also fixed, but do strongly influence the acute infection kinetics, but are just mutually non-identifiable with other parameters.

**This is a very observant point. We have now included language to this effect. Thank you.**

5. With regards to the choice of parameters to be fit in the model: Some parts of this seemed quite weird to me. Is it really possible to identify four parameters all related to the immune response? That seems very unlikely to me. And how can it be possible to fit the early growth of infection in all patients with a fixed initial condition, especially since you can never know the actual time zero (time of infection). Considering fitting this upslope alone, like any linear fit, it should require two parameters - the intercept (initial condition, independent of model parameters used here), and slope (which depends on many model parameters but only two being fit, tau and delta_S). If you are forcing a particular intercept, this is likely skewing the estimate of the slope, and hence of R0. I also found it very strange that you would fix Beta based on another study and fit tau instead. Beta is the parameter that is the most difficult to directly measure in any way and therefore seems the oddest choice to fix. There are ways to measure tau and delta_I and \delta_S and so they seem like much more natural choices. The study you cite for measuring Beta is from viral rebound after ART interruption, not acute infection, so it is very likely that Beta has a different value than in this study. And, these authors didn’t include unproductive infection, so their measured Beta would actually be Beta*tau in your model. Why not use another source to fix tau instead?

**There are many valid questions/concerns in this comment. We recognize that the model fitting here has several weaknesses. \beta and \tau are not identifiable from these data, and thus fitting either results in the same numerical values for R0, which is ultimately what matters going forward with bNAb neutralization. However, we were persuaded and changed the fitting to be for beta instead of tau (see the new figure 2).**

**Also, based on this comment, we double checked by fitting the same model with non-linear mixed effects modeling, and actually these 5 immune parameters were fairly reasonable to estimate simultaneously (RSE<50% for all). However, because trajectories are so heterogeneous, we believe better fits were achieved with individual estimates rather than mixed effects.**

**Finally, we believe estimation of infection time justifies its own paper based on the challenges of data interpretation and integration with other data sources. Here we trust the alignments to first positive from the experimental study.**

6. From the methods, it seems like the fitting was done on an individual level, so how are the population-level parameter distributions from Figure 2C generated?

**These are simply the median of parameter values across all individuals. We found it is interesting that even without addressing correlations in parameters across individual fits, we landed on a plausible viral load. This also speaks a bit to the identifiability concern in the previous comment.**

7. For the many parameters that are fixed (Table 2), just adding a citation to a paper is not really a very good explanation for why you chose this value and a reader has no way of assessing the validity of this unless they go and read all these papers. It is important to many readers whether this parameter was actually measured mechanistically or just coming from another modeling group’s model fits. Would it be possible to include a paragraph in the methods or SI explaining all these sources?

**This is a fair point. We have written fairly extensively about our parameter choices in previous works using the same model. Because this work is long already we did not recopy the discussion. We have now added a short paragraph on experimental identification that goes with the table.**

8. In the main text you jump write from talking about a deterministic model to referring to a stochastic one without even a sentence or two describing the relationship between the two.

**Good point. Perhaps this got lost in revisions, we now have a sentence to help the transition.**

9. Figure 4: It doesn’t really make sense to report that a placebo has efficacy. If 40 functional exposures per year are observed (3% annual incidence) than you must make sure this comes out of your model. If some % of patients experience natural clearance, then the observed 40 exposures comes from True exposure rate * (1-% cleared). So, perhaps there are actually 45 exposures per year

**This is very well said. We have edited language slightly to indicate we are just renormalizing so that we do not artificially boost PE when simulated burnouts do occur in placebo recipients too.**

10. Figure 5A: Why is there so much noise in these points? How big is each trial that was simulated? How long was the trial? This info should be in the caption.

**This information is now included in the caption.**

11. Figure 6D: I think the c4 figure is wrong. Doesn’t match up with Figure 5A. As potency reduction factor goes up, strains with lower breakthrough IC50 should be able to get through. And as fraction resistant goes down, same thing. But this is opposite of Figure 6. Same with c5, standard deviation in IC50

**Thank you very much for noticing this error. The axes are now flipped correctly.**

12. SI: Some equation references are not working. Figure S2 panel labels wrong (don’t agree with caption)

**We really appreciate the attention to detail. These are now fixed.**

Reviewer #2: The authors have constructed an elegant mathematical model to describe antibody-mediated HIV prevention. The model is particularly impressive because it incorporated a large amount of real-world data from studies in which broadly neutralizing antibodies to the HIV envelope protein have been infused into study participants in a effort to prevent or control HIV infection. A key question addressed in the study is whether the limited efficacy seen in the prevention studies done to date reflects a lack of breadth (ability of the antibody to neutralize many different HIV variants) or a lack of potency (ability of the antibody to neutralize at different concentrations. The authors believe that from clinical trial data they can distinguish the cause of failure. This is important because it could guide future efforts to improve efficacy. The lack of breadth could be addressed by the choice of different antibodies while the lack of potency may require efforts to improve antibody pharmacokinetics.

Overall, this is a well written and potentially important study. I have only one concern. The authors emphasize the distinction between breadth and potency but in fact they are closely related. Antibodies that lack breadth simply have low potency for neutralizing diverse strains. I am not sure how this impacts the model, but some consideration of this concept should be included at least in the discussion.

**We thank the reviewer for these comments and reading. Indeed breadth and potency can be hard to disentangle. As a result we have made the specific definitions whereby potency is essentially a characteristic of a bnab to a given challenge virus, whereas breadth is a characteristic of a bnab against the host of viral diversity in the population. This does require a notion of “resistant” which includes potency. So we are clear that we call resistant viruses those with IC50>50µg/mL, while recognizing in reality this is a continuum.**

**When we speak of breadth, we are really talking about the broadness of the distribution of IC50s. And, when we speak of potency, we are talking about a global change in neutralization regardless of the underlying distribution of IC50s. This can be accomplished by imagining a global scaling of the average IC50, though we prefer thinking about scaling all IC50s.**

**We really appreciate this comment and now include text in the discussion making this point precisely.**

Reviewer #3: The authors use a within-host infection model-based simulation strategy to develop a strategy to analyze clinical trial data from the AMP studies to gain insight into some mechanistic aspects of antibody-neutralization mediated protection. I especially liked the author's approaches of using clinical trial data for estimating parameter variability in their within-host infection models that were used for their downstream simulations, as well as using simulated clinical trial data to estimate unmeasurable mechanistic variables as a regression of measurable clinical trial outcomes. However, I do find the following major and minor concerns.

**We appreciate the reviewer’s evident close reading and valuable comments.**

MAJOR CONCERNS:

1. Fig. 3 and analyses of pharmacodynamics: In the first part of the manuscript, the authors use data from the CATNAP database for VRC01 neutralization. It is not clear how viruses with both IC50 and IC80 greater than threshold are treated (the authors mention that Hill slope is median in such cases, but how is IC50 treated?). Such cases can arise due to two different scenarios: i) virus is neutralized (e.g. to 30% at highest concentration tested), but does not reach 50%, or ii) the virus lacks certain key mutations that make it completely resistant to the antibody (i.e. 0% neutralization). The latter case corresponds roughly to the "resistant" fraction used in the latter part of the paper. Looking at Fig 3B it seems that there are no viruses that fall in the latter category -- this does not seem likely to me. I urge the authors to address this issue and state their conventions clearly in the manuscript. Looking at only IC50 and IC80 will not be sufficient to distinguish the two scenarios, and in such cases, I recommend the use of fully resistant virus (scenario ii).

**As a first point, we are now clearer about our conventions in the Methods (see ~line 500)**

**The other point in this comment is subtle and very important and we thank the reviewer for this suggestion. Indeed it was the challenge of distinguishing these two scenarios that led us to suggest a “maximum” IC50 in the first place. That is, based on the inability of the experiments to tell the difference, we wanted to allow some of the saturated IC50s to have 500ug/mL, 5000ug/mL, and so on. Thus, in the global sensitivity analysis, we performed simulations where each value of IC50=50 was instead drawn from a uniform distribution between 50 and the max IC50, the statistically simplest way to simulate this saturation effect in the experiment. We were somewhat surprised however to find that in the sensitivity analyses this maximum value wasn’t very influential on results. We suspect that is because in most cases, if a virus has IC50=50, it is likely to infect, because most PK curves in both dosing arms drop below 50 after 2-4 weeks. Thus, making IC50s = 500 instead does allow for some rare breakthroughs earlier in dosing intervals, but these events just don’t happen that often.**

**However, to highlight this decision to allow for theoretically higher IC50s in the latter half of the paper, we have included randomly sampled IC50s above the saturation level in Fig3B. This does not change median behavior.**

2. Lines 217-225: While the authors mention it in the Methods, I recommend describing in this section how the new distributions of viral sensitivity were chosen. It is not clear why the authors chose the particular values for the bimodal distribution, please explain. Also, given the second scenario in the above comment, the "resistant" IC50 centered around 10^3ug/ml would still give substantial neutralization in the early infusion cycle. Thus, I recommend the use of a higher threshold to characterize completely neutralization resistant viruses.

**We appreciate this concern but apologize because we have not opted to change all the simulations. We believe our sensitivity analysis on maximum IC50s covers this situation. We now write about these choices in line 230 as per your good suggestion.**

3. Clinical trial regression model: It is not clear if the authors have done a variable selection. It seems that the first time point viral load variables might not be adding much to the model, and it would be interesting to see how the predictions look with just the 3 other variables (protection efficacy and IC50 mean and std. dev.). I recommend the authors to explore this. Also, please use a more standard 5-fold cross validation instead of just one instance of a random 80-20 training-test split.

**We did not perform variable selection, though we have seen that leaving out VL does not really hurt estimates. However, because these data will be collected regardless, we favor leaving them in even if they only provide minimal predictive power.**

**We now perform the 5-fold cross-validation as suggested, this is a better approach, thank you. Results are updated in Fig6 and text is changed (line285).**

MINOR CONCERNS:

1. Lines 39-40: Several studies have shown that use of bnab combinations results in both increase of breadth and potency of neutralization. Thus, while the authors' statement is not incorrect, I find that the distinction between the scenarios is not a particularly important motivator. To me, a more important motivator is how can we estimate in vivo efficacy of antibody neutralization, for which an estimate of reduction of potency is paramount.

**This is a fair point, we also agree that the potency reduction factor is the more important variable to uncover in this scenario.**

2. Lines 138-141: The naive interpretation that h~1 implies a single antibody binding a single virion is likely incorrect, given that multiple trimers exist on virions and most of them need to be inactivated by antibody binding for neutralization (see e.g. Brandenberg et al Trends in Microbiol 23(12) 763-774 (2015)). While higher stoichiometry binding lead to higher slopes, heterogeneity of rate constants across sub-populations in the genetically identical viral sample (coming from glycan heterogeneity, structural dynamics, etc.) can lead to an averaging of Hill curves that can reduce the slopes (similar to Fig. 3B) and lead to h~1. Thus, I urge the authors to reevaluate this statement, and discuss it appropriately.

**We were unaware of these more nuanced explanations and instead of adding more complexity to the writing at this stage, we have removed text about the mechanistic interpretation of h=1.**

3. Fig. 3D-E: I find the lines to be too thick and/or less transparent, and find that the average curves do not visually conform to the simulations (particularly the data shown in 3E vs 3F). I recommend using thinner or more transparent lines.

**Thanks for point this out, we hope the new versions are more pleasant/interpretable.**

4. Lines 327-329: The authors can also mention that the regime in which all virions in an exposure are completely neutralized (i.e. 0 infected cells) will almost always result in R0 < 1, and is also, at least partially, captured by your model.

**Thanks, though we were trying to be as conservative about the interpretation of the model as possible, we appreciate this point, and now added a line to this effect in the discussion.**
